# Monthly Record of Deaths in the City of Buffalo

**Published:** 1859-04

**Authors:** P. H. Strong

**Affiliations:** Health Physician


### ART. VIII.—*Report of Mortality in Buffalo for the Month of Feb.*, 1859.

By P. H. Strong, M. D., Health Physician.

| DISEASES. | | No. | Males. | Females. | No Sex given. |
|---|---|---|---|---|---|
| Accidental, | | | | | |
| By Burning Fluid, | 2 | | | | |
| By Drowning, | 1 | 3 | | 3 | |
| Apoplexy, | | 2 | 2 | | |
| Convulsions, Infantile, | | 15 | 10 | 3 | 2 |
| Croup, | | 3 | 1 | 2 | |
| Cholera Infantum, | | 2 | | 1 | 1 |
| Congestion of Brain, | | 1 | 1 | | |
| Congestion of Lungs, | | 2 | 2 | | |
| Debility, from birth, | | 9 | 3 | 6 | |
| Dentition, difficult, | | 1 | 1 | | |
| Diarrhœa, | | 2 | 1 | 1 | |
| Disease of Heart, | | 3 | 3 | | |
| " Kidney, | | 1 | 1 | | |
| " Lungs, | | 1 | 1 | | |
| Dropsy in Brain, | | 5 | 1 | 4 | |
| Exposure, (infant found in street,) | | 1 | | 1 | |
| Fever, Puerperal, | | 1 | | 1 | |
| " Scarlet, | | 5 | 2 | 3 | |
| " Typhus, | | 2 | 1 | 1 | |
| Hooping Cough, | | 1 | | 1 | |
| Hypertrophy of Heart, | | 1 | 1 | | |
| Inflammation of Brain and Meninges, | | 1 | | 1 | |
| Inflammation of Larynx, | | 1 | | 1 | |
| Inflammation of Lungs, | | 11 | 9 | 2 | |
| Inflammation of Stomach, | | 2 | | 2 | |
| Inflammatlon of Veins, | | 1 | 1 | | |
| Marasmus, Infantile, | | 1 | | 1 | |
| Old Age, | | 6 | 3 | 2 | 1 |
| Ovarian Tumor and Ascites, | | 1 | | 1 | |
| Paralysis, | | 1 | | 1 | |
| Premature Birth, | | 3 | 1 | | 2 |
| Rheumatism, | | 2 | 1 | 1 | |
| Small Pox and Varioloid, | | 6 | 3 | 3 | |
| Spinal Disease, | | 1 | | 1 | |
| Still Born, | | 9 | 6 | 3 | |
| Syphilis, Secondary, | | 1 | 1 | | |
| Tuberculosis Pulmonalis, | | 10 | 7 | 3 | |
| Unknown, | | 4 | 2 | 1 | 1 |
| Total, | | 121 | 65 | 49 | 7 |

### SEXES.

| | |
|---|---|
| Males, | 65 |
| Females, | 49 |
| Sex not given, | 7 |
| Total, | 121 |

| | | |
|---|---|---|
| Died at the Almshouse, | 6 | |
| " Hospital of the Sisters of Charity, | 5 | |
| " Foundling Asylum, | 4 | 1 5 |
| " in city at large, | | 106 |
| Total, | | 121 |

| | |
|---|---|
| Of this No. there were certified by Undertakers, (9 still-born), | 45 |
| " " " by Coroner, | 2 |
| " " " by Irregular Practitioners, | 23 |
| " " " by Regular Physicians in Public Institutions, | 15 |
| " " " by Regular Physicians in Private Pra tice, | 36 |
| Total, | 121 |

AGES.

| | | | |
|---|---|---|---|
| Still-born, | 9 | Between 40 " " 50 " | 8 |
| Under 1 year | 42 | " 50 " " 60 " | 0 |
| Between 1 year and 5 years, | 26 | " 60 " " 70 " | 4 |
| " 5 " " 10 " | 6 | " 70 " " 80 " | 3 |
| " 10 " " 15 " | 5 | " 80 " " 90 " | 1 |
| " 15 " " 20 " | 3 | " 90 " " 100 " | 0 |
| " 20 years and 30 years, | 4 | " 100 " | 0 |
| " 30 " " 40 " | 6 | Unknown, | 3 |

| | |
|---|---|
| Total less than 5 years, | 78 |
| Total at 5 years and over, and unknown, | 43 |
| Total, | 121 |

NATIVITIES.

| | | | |
|---|---|---|---|
| American, | 93 | French, | 0 |
| German, | 15 | Holland, | 0 |
| Irish, | 10 | Swiss, | 0 |
| Canadian, | 1 | Prussian, | 0 |
| English, | 1 | Italian, | 0 |
| Scotch, | 0 | Country not given, | 1 |

Total, 121

Comparative Feb'y mortality for the 5 years next preceding 1849:

| | | |
|---|---|---|
| For February, 1854, | 140 | |
| " 1855, | 147 | |
| " 1856, | 111 | |
| " 1857, | 191 | |
| " 1858, | 116 | |
| Average February mortality for 5 years, | | 141 |
| For February this year, | | 121 |

